# Kinship and Care: Racial Disparities in Potential Dementia Caregiving in the United States From 2000 to 2060

**DOI:** 10.1093/gerona/glae106

**Published:** 2024-11-07

**Authors:** Kai Feng, Xi Song, Hal Caswell

**Affiliations:** Department of Sociology, Population Studies Center, University of Pennsylvania, Philadelphia, Pennsylvania, USA; Department of Sociology, Population Studies Center, University of Pennsylvania, Philadelphia, Pennsylvania, USA; Institute for Biodiversity and Ecosystem Dynamics, University of Amsterdam, Amsterdam, The Netherlands; (Medical Sciences Section)

**Keywords:** Alzheimer’s, Health disparities, Home care

## Abstract

**Background:**

Although the family plays a pivotal role in older adults’ care, there is limited research on how evolving demographic trends affect older adults’ support networks and how the trends vary by race. To fill this gap, we examine the influence of shifting family demographics on future care needs for older adults with dementia, emphasizing the unequal health and potential caregiving burdens by race in the United States.

**Methods:**

Using demographic models of kinship, we estimate the availability of potential caregivers, and dementia prevalence among one’s kin by race, kin type, and the age of a focal person from 2000 to 2060. We introduce an index called the *Dementia Dependency Ratio* to assess dementia caregiving demands at the population level, taking into account the age and kinship structure of the population.

**Results:**

Our findings suggest that Black individuals tend to have more children, grandchildren, and nieces/nephews as they age. However, Black individuals also tend to have more kin with dementia compared to their White counterparts. This elevated prevalence of dementia among Black kinship networks counterbalances the advantage of having more kin as potential caregivers. A further projection analysis suggests that the racial gap in caregiving demand within the kinship network will widen in the next 4 decades if the racial gap in dementia prevalence remains unchanged.

**Conclusions:**

These findings emphasize the urgency of reducing racial inequality in dementia prevalence rates and increasing public support for families with extended members affected by dementia. With the shrinkage of nuclear families and population aging in the next few decades, extended family members may undertake more caregiving responsibilities for dementia. We call for a kinship perspective in understanding dementia care in future research.

Family members play a crucial role in caring for older adults, particularly those with dementia. Caregivers within the kinship network, such as children, spouses, parents, siblings, and other relatives, offer a variety of assistance to family members with dementia, ranging from emotional support, transportation, housework, and personal care, to direct financial aid (1). The role of family caregiver is expected to become even more significant for older adults as the population ages (2). The availability of kin members for older adults with dementia not only correlates with the amount of informal care that a person with dementia might receive, but the absence of such kin can also indicate a greater likelihood of utilizing formal care services (3,4). Furthermore, kinship extends beyond direct or indirect support for people with dementia; it also forms an important network for family caregivers themselves, offering them mutual support and assistance (5).

The availability of family members, or kin, can differ significantly among various racial groups. Existing studies on Black–White disparities in older adults’ support networks in the United States have produced mixed results. Some research indicates that Black older adults have smaller networks of children and relatives than their White peers (6,7). Other studies suggest that the network size is not a good predictor of the amount of assistance. For instance, Oyeyemi et al. (8) found that Black older adults had more extensive assistance networks, with a higher number of family helpers, both before and during the coronavirus disease 2019 (COVID-19) pandemic, than their White counterparts. Similarly, Roth et al. (9) reported that White individuals were more likely than Black individuals to perceive lower caregiver availability. As noted in previous research, discrepancies in findings may arise from variations in network definitions and the operationalization of informal social support across studies (10). Factors such as the life stage and gender of the participants might also influence these outcomes (11).

Among dementia care literature, it is well documented that racial minorities are more likely to rely on family caregivers (3,12). Yet, few studies directly examine the racial differences in kin availability for older adults with dementia and its impact on dementia care. Using data from the 2002–2014 Health and Retirement Study, Choi et al. (3) found that among older adults over age 55 with dementia, Black individuals are less likely to have a spouse present (70.6% vs 60.9%), but have more children on average, and are more likely to coreside with at least 1 child (31.4% vs 18.3%). They also found that the absence of children strongly predicts the use of formal care services for older adults with dementia. These findings are consistent with results from the 2015 National Health and Aging Trends Study (NHATS) and the National Study of Caregiving (NSOC), which indicated that Black individuals over age 65 with dementia are more likely to receive assistance from a child or other relatives and less likely to receive support from a spouse compared to their White counterparts (13). The greater reliance on family caregivers among racial minorities may reflect cultural norms that place more value on family, distrust of the healthcare system, limited financial resources, and structural barriers to accessing services (14–16).

Another often overlooked factor in understanding the late-life family care network is the impact of long-term demographic changes (17,18). These changes significantly influence the size and composition of the available family care network for older adults. For instance, the increase in life expectancy has substantially extended the anticipated number of years an individual might spend with their grandchildren (19). High fertility rates create a larger “sandwich” generation in many countries, where individuals find themselves squeezed between caring for dependent children and frail older parents (20). The complex interplay of changing fertility and mortality rates suggests that an individual’s family structure and kinship network, both in size and composition, can vary depending on their age and the specific period in which they live. Research on family support networks conducted 30 years ago may yield different findings compared to contemporary studies, simply because family structures and kinship networks evolve over time. With the growing accessibility of multigenerational data (21) and advances in methodology (22), researchers have only recently begun to examine racial differences in kinship size and composition (23,24). With a few exceptions (25), most studies have centered on results for a single year. Research exploring how shifting demographic trends influence racial disparities in older adults’ support networks, and how these differences may change over time, remains understudied (17).

Children and spouses are often identified as the primary caregivers in the dementia caregiving literature, but the role of extended kin has long been ignored (26). Recent studies showed that relatives other than children and spouses also play a significant role in providing care for older adults with dementia (4,27–29). For example, Wolff et al. (29), utilizing data from the NHATS and NSOC, found that extended kin represents 16.7% of caregivers offering significant assistance with healthcare activities (both care coordination and medication management) and account for 23.9% of those providing some help (in either care coordination or medication management). Using the Health and Retirement Study, Friedman et al. (4) showed that relatives other than children or spouses provided 40.7 hours in the past month on average to adults above age 70 with cognitive impairment but not dementia, and 70.8 hours to adults with probable dementia, numbers comparable to the hours that the son of older adult provides (4). This trend is part of a broader pattern: older adults in need of long-term care, especially those who are single or childless, frequently report receiving informal support from other relatives or nonrelatives (30). As the proportion of the single or childless demographic grows, there is an anticipated rise in reliance on extended families (31), particularly among the Baby Boomer generation (2), and in times of crises like the COVID-19 pandemic (32). Such patterns highlight the need to examine changes in the broader kinship network over time, along with their implications on older adult care (26).

In this study, we adopt new demographic models to investigate the interrelations between changing family demography and future family care networks for older adults with dementia, with a focus on the Black and White differences in the United States. We focus on dementia care in our study because, in comparison to other forms of older adults’ care, dementia care is notably more intensive and typically involves a larger number of caregivers (33,34). Our emphasis on the disparity between Black and White populations is informed by findings that Black individuals consistently exhibit a higher prevalence of dementia than Whites (35–37). Furthermore, Black caregivers typically contend with more intense caregiving responsibilities and face greater financial challenges compared to their White peers (38,39).

Specifically, we project the kinship network’s size and composition for a randomly chosen individual, termed as Focal, by race, kin types, and the Focal’s age. We provide estimates for a wide range of kin types, including siblings, grandchildren and great-grandchildren, aunts, uncles, nephews and nieces, and cousins. To ease discussion, we present the number of available kin by kin type for Focal aged 65 and 85 from 2000 to 2060. Applying race- and age-specific dementia prevalence to the kinship network, we also estimate the possibility of having at least one kin with dementia by race, kin types, and the age of Focal. Because the Black–White inequality in family caregiving is, in part, influenced by both the unequal demand for caregiving and the availability of kin who can serve as caregivers, we quantify dementia caregiving needs at the group level using a new dementia dependency index. The index calculates the ratio of the Focal’s number of kin with dementia to their working-age kin without dementia. This index is a function of both the Focal’s age and kinship structures.

## Data and Method

### Define Kin

Defining kin or family is challenging with increasing family diversity and cultural variations (40). In this study, we focus exclusively on blood-related (consanguineal) ties but include a broad range of kin types including children, parents, grandchildren, grandparents, great-grandparents, great-grandchildren, siblings, nephews and nieces, aunts and uncles, and cousins. Supplementary Figure 1 illustrates a kinship diagram based on this descent. We begin with female rates for mortality and fertility to estimate Focal’s kinship network from matrilineal descent. To approximate the total number of kin, we adopt a method by Goodman, Keyfitz, and Pullum (41), which assumes identical rates for males and females (see Author Note 1). A recent study that compared kin counts from formal kinship models using an approximation approach with empirical measurements derived from Swedish registry data demonstrated a high degree of precision of the formal demographic method (22).

The main goal of the study is to provide estimates of the availability of kin for older adults with dementia by race, age, and type of kin. Our analytical approach assumes that the extent of kin involvement in caregiving varies according to the closeness of the kin relationship. For instance, children of an older adult with dementia will likely be more involved in caregiving than cousins of the adult. Whenever possible, we present results by specific kin types rather than combining them. However, in constructing the dementia dependency index, we aggregate all kin types because of the increasing relevance of extended kin, as previously discussed. In Supplementary File, we offer 2 alternative measurements: (1) including only parents and children (combining siblings and the Focal), and (2) including all kin types but assigning varying weights based on genetic closeness, as proposed by Murphy (42).

### Kinship Model With Time-Varying Rates

For our projection analysis, we employ recently developed demographic models of kinship, which have been extensively detailed in Caswell (43) and Caswell and Song (34). This approach utilizes time-varying age-specific demographic rates to derive various summary statistics of kinship networks. The fundamental methodology underlying this approach is to treat each type of kin of the Focal as a distinct population and to project these populations from one age of the Focal to the next using matrix population models. This approach has been previously applied to investigate kin loss (44), racial disparities in exposure to unemployment (45), and dementia caregiving demands in China (46). Although recent research has successfully derived kinship networks directly in regions with high-quality administrative register data, such as the study by Kolk, Andersson, Pettersson, and Drefahl (47) for Sweden, analytical mathematical models or simulations remain essential for projecting future kinship structures. We employed the recently introduced R package, *DemoKin*, for our calculations (48).

### Dementia Prevalence Among Kinship Network

Let *k*(*x*, *t*) represent the age distribution of a specific type of kin relative to a Focal individual aged *x* at time *t*. If *Ψ* is a vector containing age-specific prevalence of dementia, then the expected number of kin with dementia at age *x* of Focal at time *t* is:


$$\begin{array}{l} y\left(x,t\right)=\;\Psi {\left(t\right)}^{T}k\left(x,\;t\right) \end{array}$$ (1)

We calculate the probability that Focal, at age *x* and time *t*, has at least 1 kin of a specific type with dementia based on the number of kin with dementia. This calculation employs a Poisson approximation, similar to the approach used in Song and Mare (19) and Song, Campbell, and Lee (49). If the expected number of kin with dementia at time *t* is *y*(*t*), under the Poisson assumption, the probability of having at least one such kin is as follows:


$$\begin{array}{l} \;P\left(at\;least\;one\;kin\;with\;dementia\right)=\;1-e^{-y\left(t\right)} \end{array}$$ (2)

### Dementia Dependency Ratio

We define a dementia dependency ratio (DDR) as the ratio between the number of kin with dementia of Focal at age *x* and the number of working-age (see Author Note 2) kin without dementia of Focal at age *x*:


$$\begin{array}{lll} DDR\left(x,\;t\right) & =\frac{kin\;with\;dementia}{kin\;without\;dementia\;aged\;\;16-64}\; & =\frac{y\left(x,\;t\right)}{{\left(1-\;\Psi_{16-64}\right)}^{T}k\left(x,\;t\right)} \end{array}$$ (3)

Our index, which factors in the kinship structure, is an improvement over the traditional demographic age dependency ratio or caregiver support ratio, which primarily relies on the age distribution of the total population but ignores family relationships among individuals. The DDR serves as an indicator of the caregiving demands that working-age individuals without dementia face when supporting family members with dementia. For example, a DDR of 0.1 indicates that, on average, there are 10 working-age kin members without dementia available to potentially provide support for each person with dementia, depending on how kinship is defined. A similar index was previously proposed by Wolf (50) and Tu, Freedman, and Wolf (51). Wolf (50) highlighted that the traditional population-based dependency ratio and the kinship-based dependency ratio delineate 2 distinct extremes of dependency: The population-based dependency ratio suggests a completely collective system where all working-age individuals equally share the responsibility of supporting the older population. Conversely, the kinship-based index implies a fully private support system in which the kinship network bears the sole responsibility for caring for their older relatives.

In our main results, we present the DDR that includes all kin types based on the kinship network defined above and in Supplementary File. However, it is reasonable to expect that, for example, a cousin would be less involved in helping Focal’s parents with dementia compared to Focal’s siblings. We provide 2 alternative measurements in Supplementary File with different definitions of kinship networks. The first measurement includes only the Focal’s parents and siblings, that is, all children of the Focal’s parents, in the calculation of DDR. Following the approach of Murphy (42) in his study of Britain, the second measurement keeps all the kin types previously defined but assigns a distinct weight to each based on genetic closeness to the Focal individual. For instance, parents, children, and siblings share half of their genes with the Focal, whereas aunts, uncles, nephews, nieces, grandparents, and grandchildren share a quarter of their genes with the Focal. Great-grandparents, great-grandchildren, and cousins share one-eighth of their genes with the Focal. Accordingly, we have allocated weights of 1, 0.5, and 0.25, respectively, to mirror the degree of closeness between the Focal and these various kin types.

The DDR is an age-dependent measurement because, at each age of the Focal, she may have a different number of relatives with and without dementia. In the next section, we create an aggregate burden index to measure the race-specific dementia burden at the population level.

### The Dementia Caregiving Demands at the Population Level

The vector *k*(*x*, *t*) gives the age distribution of the kin, of type *k*, of Focal at age *x*. Thus, the dementia prevalence and the DDR capture the expected caregiving demands of dementia within a family. A population can be conceptualized as a collection of Focal individuals characterized by an age structure, represented by *n*(*t*). The overall kinship structure in the population can be weighted by taking an average over this age distribution.

We define the proportional age distribution as follows:


$$\begin{array}{l} w\left(t\right)=\frac{n\left(t\right)}{∥n\left(t\right)∥} \end{array}$$ (4)

Then the age-weighted, population dependency ratio is expressed as:


$$\begin{array}{l} DDR\left(pop\right)=\underset{x}{\sum }\;w_{x}\left(t\right)DDR\left(x,\;t\right) \end{array}$$ (5)

This quantity, as a singular numeric index, represents the expected DDR for an individual randomly chosen from the population in a single year. Specifically, it illustrates the ratio between Focal’s kin members with dementia to Focal’s kin members without dementia.

We calculate this index for all years between 2010 and 2060 for both racial groups (see Author Note 3).

### Vital Statistics and Population Projection

We obtained period estimates of age-specific fertility rates by race from Heuser’s (52) fertility table for the years 1917 to 1980 and from the National Vital Statistics Reports for the years 1981 to 2018. Heuser compiled fertility tables produced by the National Institute of Child Health and Development but corrected for undercounts and age misreporting. The fertility rates are tabulated for ages 14 to 49. The original Heuser’s calculations include fertility tables through 1973 but the Office of Population Research at Princeton University updated those tables for years between 1974 and 1980 (see Author Note 4). The National Vital Statistics provide fertility rates in 5-year age groups for ages 10 to 49. We assume the fertility rate is zero for ages that are beyond the observed age range.

We obtained period estimates of age-specific survival rates by race from the U.S. Life Table and National Vital Statistics Reports published by the U.S. Census Bureau. For years when only abridged life tables were provided, we employed linear interpolation to estimate single-age estimates of *l*_*x*_. Mortality data from years before 1996 were limited to ages between 0 and 85. We thus extrapolate the mortality curve for older ages up to age 100 using the Kannisto model. We use only female rates for mortality and fertility to estimate Focal’s kinship network. We use the 2017 National Population Projections Data Sets from the U.S. Census Bureau. The data provide projected age-specific fertility rates by race for women aged 14 to 54 and age-specific mortality rates by race for both men and women aged 0 to 100, spanning from 2017 to 2060. We convert the age-specific mortality rates to age-specific survival rates (ie, *l*_*x*_ in the life table). We also derive the age distribution by using population projections for every single year of age and race from 2016 to 2060.

### Dementia Prevalence

We derive the age-specific dementia prevalence rate by gender and race from the findings of Hudomiet et al. (53), who estimated this prevalence based on data from the Health and Retirement Study. Dementia prevalence estimates are available every other year from 2000 to 2016. For gap years when estimates are unavailable, we impute the data using the dementia prevalence rate from the preceding year. We first obtain dementia prevalence rates by race in seven 5-year age groups (65–69, 70–74, 75–79, 80–85, 86–89, 90–94, 95–100), covering the age range from 65 to 100 years. Next, we apply a linear interpolation to estimate dementia rates for each year within the age range. Dementia prevalence is assumed to be zero before the age of 65. All types of dementia are progressive; nevertheless, distinguishing the stage of dementia and the related amount of care based on prevalence rate estimates is beyond our focus. To address this issue, we assume that the severity is consistent across groups. In our projection analysis, we further assume that the race- and age-specific dementia prevalence rates remained constant at the levels observed in 2016 from 2017 to 2060.

Yet, it should be noted that recent studies indicate an overall decline in dementia prevalence (54) and a narrowing gap between Black and White males (53). To the best of our knowledge, no study has been conducted on projecting future racial disparities in dementia prevalence. The only study we identified that projects dementia cases by race assumes that the race-specific dementia prevalence rates remain constant at their 2014 levels (36). We follow the same strategy in estimating the probability of having at least one kin with dementia (by kin type) and in calculating the DDR index. However, we incorporate a counterfactual analysis to address the question of what would occur if the dementia prevalence rates were identical for both Black and White populations.

## Results

**Figure 1.**
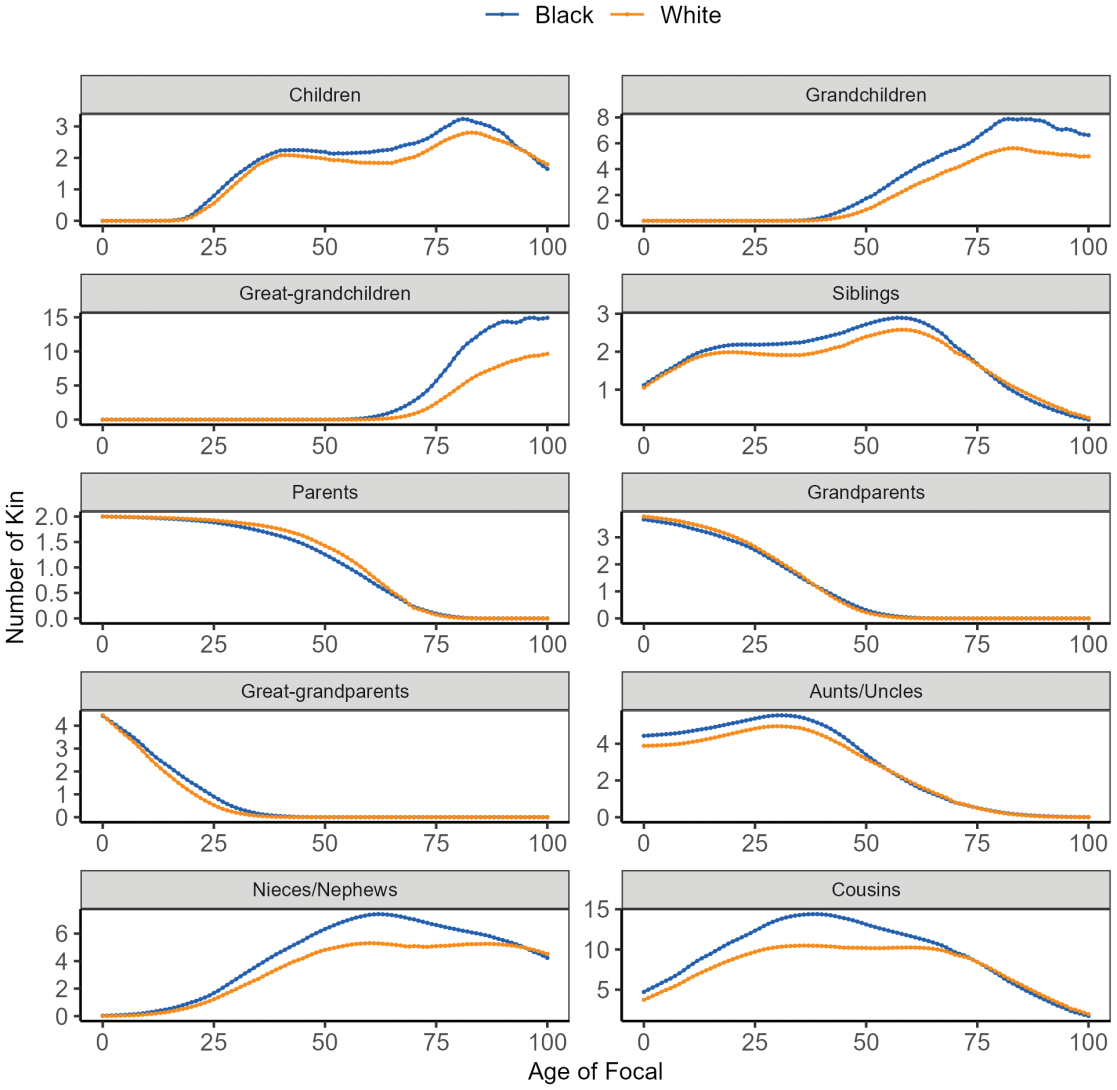
Expected number of kin of various kinds as a function of the age of focal in 2016. *Sources*: National Vital Statistics Reports, 1996–2017; Vital Statistics of the United States, 1960–1995; Vital Statistics of the United States (abridged life table), 1946–1959; United States Life Tables and Actuarial Tables, 1939–1941; United States Life Tables for 1900–1902, 1901–1910, 1909–1911, 1919–1921, 1920–1929, and 1929–1931; Fertility Tables for Birth Cohorts by Color: United States, 1917–1980; National Vital Statistics Reports for 2015 and 2018; 2017 National Population Projections Data Sets.

Figure 1 illustrates variations in kinship size by race and kin type for individuals of different ages in the year 2016. The observed racial differences reflect the historical disparities in fertility and mortality rates between Black and White populations. Higher fertility and an earlier age at childbearing tend to yield a greater number of relatives, whereas higher mortality leads to a smaller kinship network. Notably, Black individuals tend to have more children, grandchildren, great-grandchildren, aunts/uncles, nieces/nephews, and cousins compared to their White counterparts.

The curves representing the number of parents, grandparents, and great-grandparents resemble survival curves because the maximum numbers of biological parents, grandparents, and great-grandparents are fixed. We also observe that the overlapping lifetime between individuals and their parents and grandparents is longer for White individuals than Black individuals, mainly due to the lower mortality rates of the White population. By contrast, the overlapping lifetime between individuals and their great-grandparents is higher for Black individuals than White individuals. A possible explanation is that Black families tend to have childbearing at younger ages and thus are more likely to have living great-grandparents. The higher fertility among Black families also explains why Black individuals tend to have more siblings than their White counterparts before the age of 74. However, this trend reverses after 74, primarily due to the difference in mortality rates between the 2 populations.

**Figure 2.**
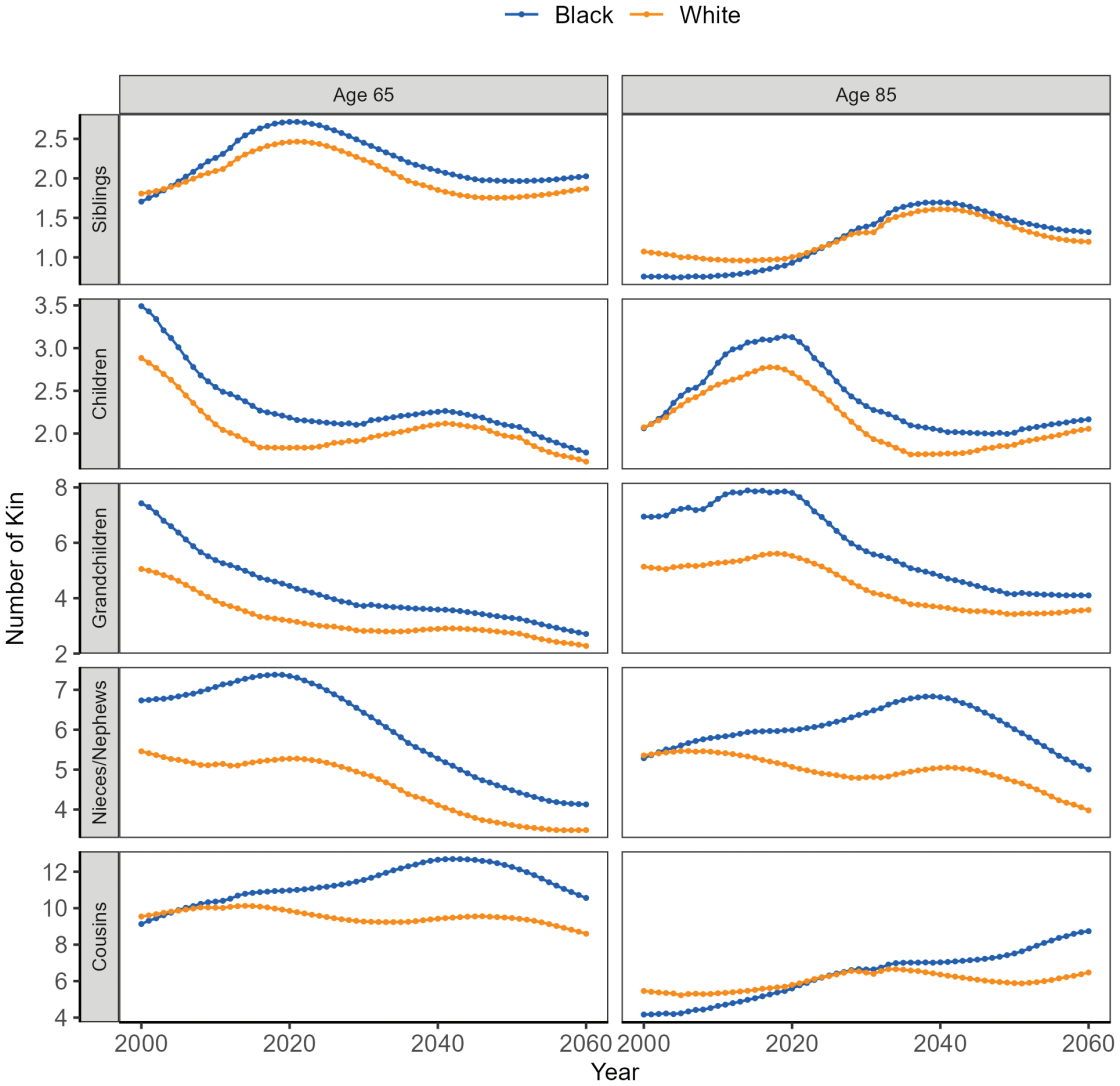
The changing expected number of kin of various kinds for individuals at the ages of 65 and 85 by race from 2000 to 2060. *Sources*: National Vital Statistics Reports, 1996–2017; Vital Statistics of the United States, 1960–1995; Vital Statistics of the United States (abridged life table), 1946–1959; United States Life Tables and Actuarial Tables, 1939–1941; United States Life Tables for 1900–1902, 1901–1910, 1909–1911, 1919–1921, 1920–1929, and 1929–1931; Fertility Tables for Birth Cohorts by Color: United States, 1917–1980; National Vital Statistics Reports for 2015 and 2018; 2017 National Population Projections Data Sets.

A crucial question in understanding the support systems available for older adults is assessing the number of available family members whom they can rely on. Figure 2 presents the projected count of available kin by race and kin type for individuals aged 65 to 85 from 2000 to 2060. We have excluded the numbers for parents, grandparents, great-grandparents, and aunts/uncles, under the assumption that individuals in these categories are at a very old age and can provide limited assistance themselves.

From 2000 to 2060, Black individuals at older ages are projected to have a higher number of children, grandchildren, and nieces/nephews. Some of the differences observed are substantial. For instance, in 2020, Black individuals at age 85 are expected, on average, to have 2 more grandchildren and 1 more child than White individuals of the same age. In contrast, White individuals at the age of 85 are projected to have more siblings than their Black counterparts from 2000 to 2025, reflecting the racial differences in mortality patterns. However, this trend is expected to reverse after 2025, with Black individuals at age 85 surpassing White individuals in the number of siblings. Similarly, in 2025, Black individuals at age 85 are projected to have more cousins than their White counterparts. The changes in the number of kin for individuals in old age do not follow a straightforward, linear trajectory. Instead, they exhibit fluctuations indicative of dynamic changes in kinship structures, driven by the interplay between fertility and mortality. For example, the peak in the number of children earlier in the century reflects the trend of increased fertility rates Post–World War II, indicating that old-age individuals in this period were parents of the Baby Boomer generation.

**Figure 3.**
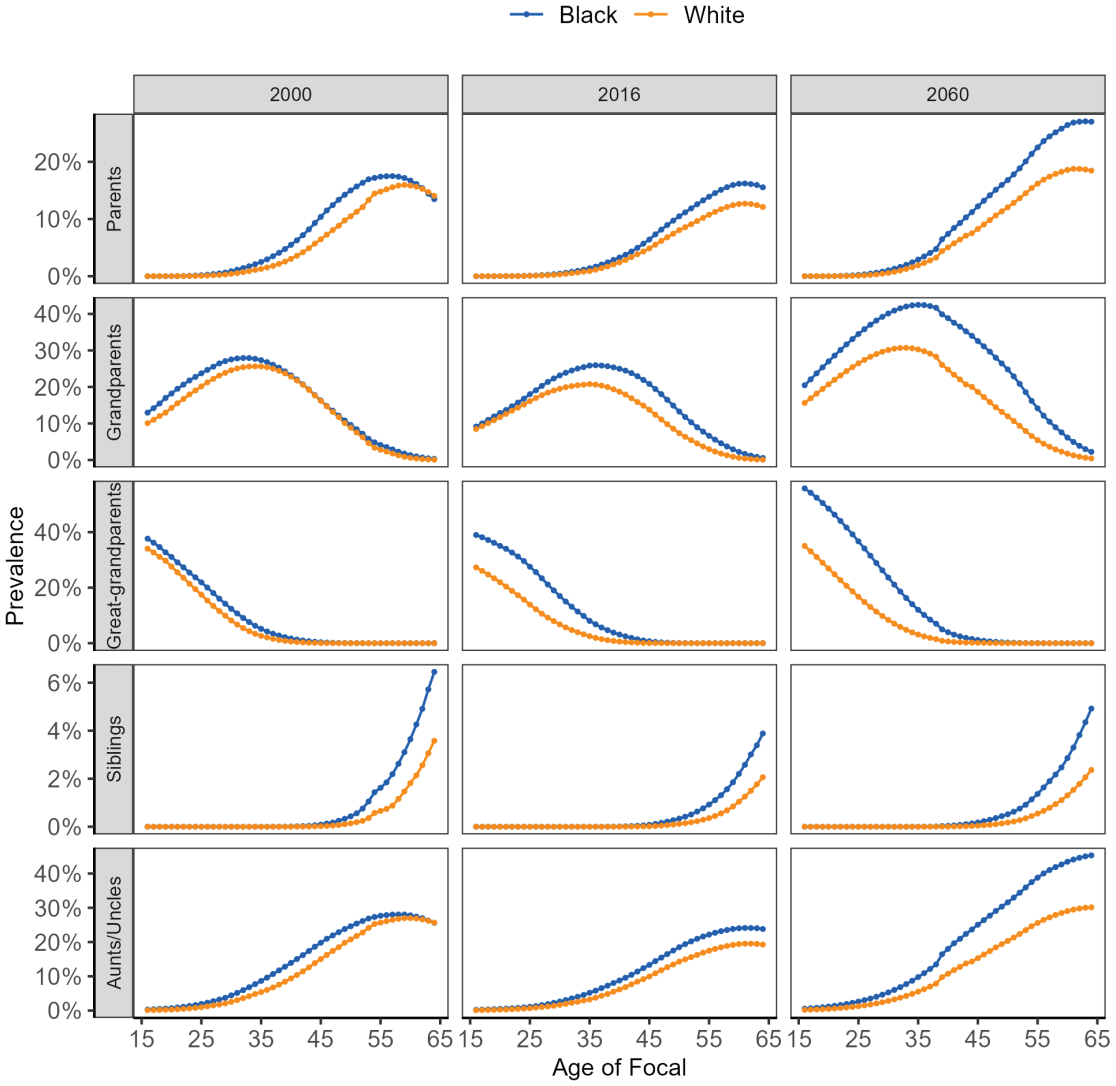
The prevalence rate of dementia for various kinds of kin by race and age of focal in 2000, 2016, and 2060. *Sources*: National Vital Statistics Reports, 1996–2017; Vital Statistics of the United States, 1960–1995; Vital Statistics of the United States (abridged life table), 1946–1959; United States Life Tables and Actuarial Tables, 1939–1941; United States Life Tables for 1900–1902, 1901–1910, 1909–1911, 1919–1921, 1920–1929, and 1929–1931; Fertility Tables for Birth Cohorts by Color: United States, 1917–1980; National Vital Statistics Reports for 2015 and 2018; 2017 National Population Projections Data Sets; Health and Retirement Study, 2000–2016 (53).

Next, we examine the prevalence of dementia among Focal’s kinship network by the age of Focal (16 to 64) for the years 2000, 2016, and 2060 in Figure 3. Note that the prevalence estimate here refers to the probability of having at least one kin member with dementia. We only present the estimates for Focal’s parents, grandparents, great-grandparents, siblings, aunts, and uncles. For projections to 2060, we assume a constant dementia prevalence rate obtained from year 2016. In nearly all kin types and across all years, an average Black individual has a higher probability of having kin with dementia compared to an average White individual. Moreover, the probability curve for Black individuals peaks earlier than that for White individuals, suggesting that Black individuals may face a greater likelihood of having kin with dementia at relatively younger ages than their White counterparts.

The probability of having at least one kin member with dementia shows a decline from 2000 to 2016, before experiencing an increase again by 2060. The reduction from 2000 to 2016 is associated with the decline in dementia prevalence rate, particularly among the White population. Nonetheless, by 2060, the probability of having at least one kin with dementia is projected to increase for both Black and White populations. This trend aligns with the anticipated decrease in mortality and increase in life expectancy observed across both racial groups. As a result, even if the prevalence of dementia remains at the 2016 level, changes in kinship structures could drive an increase in dementia prevalence within kinship networks.

Individuals at certain ages are likely to have close kin, such as siblings, parents, and grandparents, with dementia. For example, Black individuals at age 60 have a 16% chance of having at least one parent with dementia in 2016, which is 3% higher than White individuals, and this is projected to increase to 26% in 2060, 7% higher than White individuals. Overall, between 2016 and 2060, the probability of having kin with dementia increases substantially for both racial groups. With time, the racial gap in nearly all these different types of kin dementia prevalence rates will widen.

**Figure 4.**
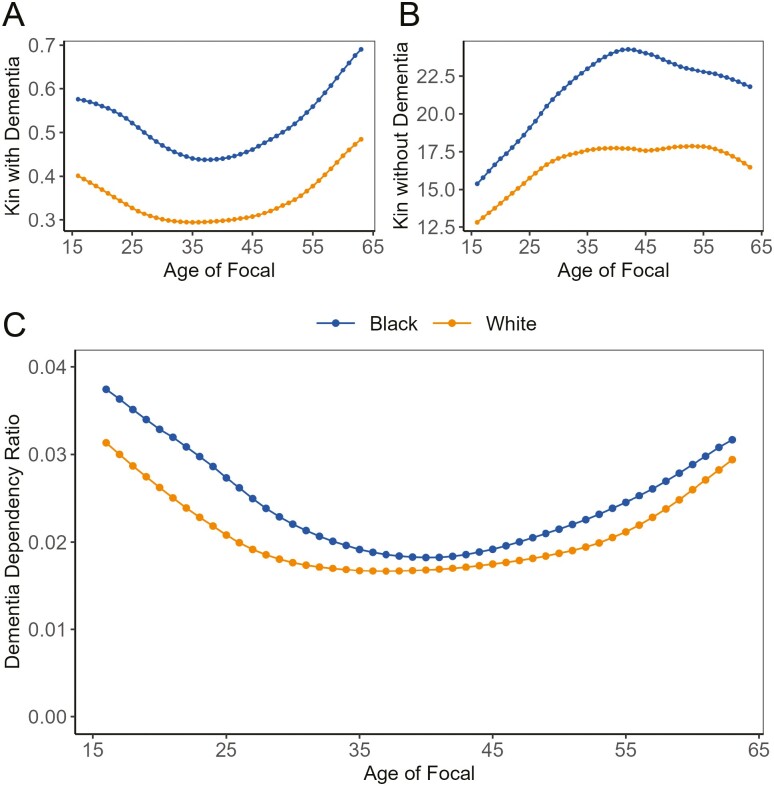
(A) The number of kin with dementia by race in 2016; (B) The number of kin aged 16–64 without dementia by race in 2016; and (C) The dementia dependency ratio as a function of the age of focal by race in 2016. *Sources*: National Vital Statistics Reports, 1996–2017; Vital Statistics of the United States, 1960–1995; Vital Statistics of the United States (abridged life table), 1946–1959; United States Life Tables and Actuarial Tables, 1939–1941; United States Life Tables for 1900–1902, 1901–1910, 1909–1911, 1919–1921, 1920–1929, and 1929–1931; Fertility Tables for Birth Cohorts by Color: United States, 1917–1980; National Vital Statistics Reports for 2015 and 2018; 2017 National Population Projections Datasets; Health and Retirement Study, 2000–2016 (53).

Figure 4C presents the age-specific DDR and its 2 components in 2016: Focal’s number of kin with dementia (Figure 4A) and Focal’s number of working-age kin without dementia (Figure 4B).

Figure 4A shows a U-shaped relationship between Focal’s age and number of kin with dementia. At the age of 16, a Black individual is expected to have 0.58 kin members with dementia, whereas a White individual is expected to have 0.40 kin members with dementia. For both Black and White individuals, the lowest number of kin with dementia occurs in their mid-30s (age 38 for Black individuals and 35 for White individuals). As individuals age, the number of kin with dementia increase for both groups. Figure 4B shows an almost reversed trend: individuals have a declining number of working-age kin without dementia after their mid-50s (55 for Black and 57 for Black). At any given age, from 16 to 64, a Black individual has more kin with dementia and more kin without dementia than a White individual. The age-specific DDR is derived from the ratio of kin with dementia of Focal at a certain age to kin aged between 16 and 64 without dementia of the Focal at that age. A higher DDR value indicates that, on average, an individual has a higher number of relatives with dementia and relatively fewer working-age kin without dementia. The age-specific DDR shows a U-shaped pattern, highlighting varying caregiving demands across different age groups. Individuals younger than 25 or older than 60 face higher dementia caregiving demands compared to those aged between 25 and 60. Furthermore, Black individuals show a consistently higher DDR across ages 16 to 64.

In our final analysis, we apply weights to the age-specific DDR based on the age structure of the population. Given that racial and ethnic minorities have a younger age structure than the White population in the United States, we derive a race-specific dementia caregiving demand index that accounts for the different age compositions of the Black and White populations.

**Figure 5.**
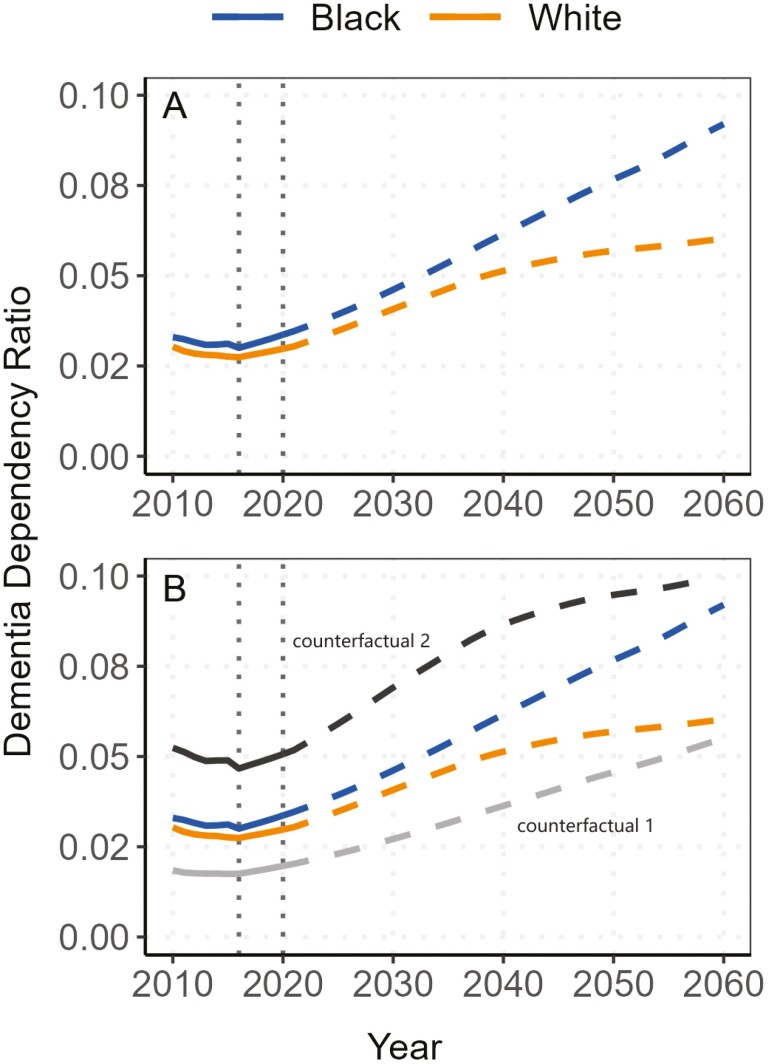
Estimated dementia dependency ratio between 2010 and 2020 and projected dementia dependency ratio from 2021 to 2060. *Sources*: National Vital Statistics Reports, 1996–2017; Vital Statistics of the United States, 1960–1995; Vital Statistics of the United States (abridged life table), 1946–1959; United States Life Tables and Actuarial Tables, 1939–1941; United States Life Tables for 1900–1902, 1901–1910, 1909–1911, 1919–1921, 1920–1929, and 1929–1931; Fertility Tables for Birth Cohorts by Color: United States, 1917–1980; National Vital Statistics Reports for 2015 and 2018; 2017 National Population Projections Datasets; Health and Retirement Study, 2000–2016 (53). In Counterfactual 1, we maintained kinship differences but equalized dementia rates to match White individuals. In Counterfactual 2, we equalized kinship structures to the White, but let dementia rates vary by race. After the first vertical dashed line in 2016, dementia rates reflect the observed values observed in 2016. Beyond the second vertical dashed line in 2020, the vital statistics are based on projections.

Figure 5A illustrates the index of the weighted DDR for both Black and White populations from 2010 to 2060. We estimate the DDR by individuals’ race and age and apply weights derived from the year-specific population age structure for each racial group to obtain the population-average DDR. Two immediate observations emerge from the results: First, the weighted DDR is projected to increase for both Black and White populations from 2010 to 2060. Second, the Black population consistently shows a higher level of DDR, and the racial gap in this ratio is also widening over time.

The observed racial gap in the DDR is a joint effect of disparities in both kinship and population structure, as well as differences in dementia prevalence rates between races. To isolate the impact of each contributing factor, we conduct 2 counterfactual analyses, the results of which are showcased in Figure 5B.

In the first counterfactual analysis, we retained the original differences in kinship structure for Black and White populations but fixed the prevalence rate of dementia for both groups to be the same as White individuals. After this adjustment, the DDR for the Black population notably decreased. From 2010 to 2060, the DDR for Black individuals, under this first counterfactual scenario, remained lower than that of the White population, but the racial gap gradually converged over time.

In the second counterfactual analysis, we assumed that both Black and White populations shared the same kinship structure, fixed at the level of the White population, while allowing the dementia prevalence rates to vary by race. The results revealed that applying the White kinship structure to the Black dementia prevalence rate led to an even higher DDR than using the White kinship structure with the White dementia prevalence rate.

## Discussion

The availability of family caregivers for older adults with dementia plays a pivotal role in predicting patterns of care utilization, care transitions, and care costs (3). In this study, we contribute to the existing literature by investigating the relationship between changing family demography and future available family care networks for Black and White older adults in the United States across 6 decades from 2000 to 2060. We estimate the availability of potential caregivers, varying by race, kin type, and the age of the Focal. Our findings suggest that Black individuals tend to have more children, grandchildren, and nieces/nephews as they age. This pattern is expected to persist, even if the racial gap in fertility will diminish in the next few decades. As a result, Black older adults in need of dementia care may have access to a larger network of potential caregivers. However, due to the higher prevalence of dementia within Black kinship networks, a larger kinship network might lead to a greater caregiving need rather than increased care support for Black families, as indicated by a higher DDR among the Black population compared to the White population.

The counterfactual analyses based on the DDR yielded 2 key findings. First, kinship structure in the White population tends to be older than that in the Black population. This pattern suggests that the familial and kinship networks among White individuals are characterized by a higher proportion of older members. This phenomenon may have implications for caregiving, support systems, and the family safety net that are influenced by the age compositions of one’s close relatives. In other words, the demographic burden driven by births and deaths within families is greater for White families than Black families. Second, the racial disparity in the weighted DDR is primarily driven by the differences in dementia prevalence rates between the racial groups. This pattern suggests that the racial gap in dementia prevalence significantly affects the overall caregiving demands caused by dementia on families. Black families face a greater need for dementia caregiving despite having a bigger kinship size and younger kin members than White families.

Our study emphasizes the urgency of addressing the racial gap in dementia prevalence rates. Black individuals have higher risks of dementia, are less likely to be formally diagnosed with dementia, and less likely to use paid help or nursing home (14,55). One consequence of the high prevalence among the Black population is a higher likelihood of having more relatives living with dementia. Black individuals may be more likely than White individuals to provide care for multiple older adults at the same time during their lifetime (56). The high dementia prevalence among Black kinship networks may exacerbate the impact of structural barriers and socioeconomic disadvantages on dementia care faced by the Black population. Future studies should investigate whether racial differences in dementia prevalence among their kinship networks contribute to differences in caregiving demands, as well as the potential impact on financial and workplace security. Policies should also strengthen public support for caregivers who may contend with caregiving tasks from multiple family members.

Our study based on formal demographic models and projections also addresses the lack of empirical evidence on kin and caregiving. Despite the increasing importance of extended kin as dementia caregivers (3,4,28), very few survey data collect information of which, when, how, and why extended family members serve as family caregivers (26). Public policies also often fail to recognize extended family members as family caregivers. For example, the Family and Medical Leave Act coverage excludes extended family members such as siblings, aunts, uncles, and other nontraditional family relations (57). More evidence-based research and policies are needed to support nontraditional family caregivers, who are undertaking an increasing share of caregiving responsibilities as nuclear families diminish in size.

We acknowledge several potential limitations of our analyses. First, we have exclusively focused on biological kin relationships and have not explored stepfamilies and kin from the spouse’s side. The complexity and diversity of family, including the definition of who can be considered as family members, are becoming crucial aspects to consider when studying the older adults’ support network (26,58,59). Another limitation is that we focus on the population average, but demographic patterns can vary significantly across socioeconomic status. For example, individuals with college degrees are more likely to have smaller family networks but are also more likely to be married (24). However, the influence of educational differences in kinship networks is less pronounced compared to the variation in kinship networks by one’s age (23). Additionally, our study has primarily concentrated on the differences between Black and White populations, without examining the within-group heterogeneity. For instance, research has revealed variations in family ties, with Black individuals in the South displaying stronger family bonds compared to those in the North (60). Furthermore, our study is limited to Black and White populations and does not account for a full range of variations across race, ethnicity, and nativity. Recent studies indicate that immigrant caregivers often engage in more time-intensive caregiving (61), and caregivers of foreign-born older adults with dementia have reported lower levels of psychological well-being (62). Last, while we offer estimates of the available kinship network by kin type, age of the Focal, and race, we do not estimate the actual caregiving provided by kin, whether it be physical, emotional, or financial support. Prior research suggests that the positive association between the size of kinship networks and the presence of actual caregivers for adults with activity limitations has a ceiling effect, plateauing at approximately 2 caregivers (24). It is possible that this relationship changes from period to period and over one’s life course, considering the composition and size of kinship networks are constantly changing over time and throughout different stages of life. We leave these important modifications and extensions for future research.

## Supplementary Material

Supplementary data are available at *The Journals of Gerontology, Series A: Biological Sciences and Medical Sciences* online.

glae106_suppl_Supplementary_Material

## Author Notes

1. This method approximates the number of children as double the count of daughters, grandchildren as quadruple the count of granddaughters, great-granddaughters multiplied by 8, mothers by 2, grandmothers by 4, great-grandmothers by 8, sisters by 2, nieces and aunts by 4 each, and cousins by 8.

2. Same with the population-based dependency ratio, we define the working-age population as individuals aged between 16 and 64. This is also the age range that includes the majority of caregivers. Extending the age range to between 8 and 75 does not substantively affect our conclusions (see Supplementary File).

3. We picked the year 2010 as the starting year as race-specific population age structure data are not available for earlier years in the 2017 National Population Projections Data.

4. Heuser’s U.S. Cohort and Period Fertility Tables can be downloaded from the OPR data archive:https://oprdata.princeton.edu/Archive/CPFT/ (accessed September 30, 2023).

## References

[1] Friedman EM , Kennedy DP. Typologies of dementia caregiver support networks: a Pilot Study. Gerontologist. 2021;61(8):1221–1230. 10.1093/geront/gnab013 33585929 PMC8599268

[2] Redfoot D , Feinberg L, Houser A. Baby Boom and the Growing Care Gap: A Look at Future Declines in the Availability of Family Caregiver. Vol. 12. Washington, DC: AARP Public Policy Institute; 2013. https://www.aarp.org/home-family/caregiving/info-08-2013/the-aging-of-the-baby-boom-and-the-growing-care-gap-AARP-ppi-ltc.html. Accessed January 28, 2024.

[3] Choi H , Heisler M, Norton EC, Langa KM, Cho T-C, Connell CM. Family care availability and implications for informal and formal care used by adults with dementia in the US. Health Affairs (Project Hope). 2021;40(9):1359–1367. 10.1377/hlthaff.2021.00280 34495713 PMC8647567

[4] Friedman EM , Shih RA, Langa KM, Hurd MD. US prevalence and predictors of informal caregiving for dementia. Health Affairs (Project Hope). 2015;34(10):1637–1641. 10.1377/hlthaff.2015.0510 26438738 PMC4872631

[5] Cleary JL , Manalel JA, Ashida S, Marcum CS, Rewley J, Koehly L. Interpersonal correlates of dementia caregivers’ emotional support networks: considering family history. Res Aging. 2022;44(5–6):405–413. 10.1177/01640275211026919 34372731 PMC12376061

[6] Barnes LL , Mendes de Leon CF, Bienias JL, Evans DA. A longitudinal study of Black–White differences in social resources. J Gerontol B Psychol Sci Soc Sci. 2004;59(3):S146–S153. 10.1093/geronb/59.3.s146 15118020

[7] Ajrouch KJ , Antonucci TC, Janevic MR. Social networks among Blacks and whites: the interaction between race and age. J Gerontol B Psychol Sci Soc Sci. 2001;56(2):S112–S118. 10.1093/geronb/56.2.s112 11245365

[8] Oyeyemi DM , Lin I-F, Wang H, et al Changes in late-life assistance networks for Black and White older adults during the COVID-19 pandemic. J Am Geriatr Soc. 2023;71(11):3574–3583. 10.1111/jgs.18547 37587898 PMC11654626

[9] Roth DL , Haley WE, Wadley VG, Clay OJ, Howard G. Race and gender differences in perceived caregiver availability for community-dwelling middle-aged and older adults. Gerontologist. 2007;47(6):721–729. 10.1093/geront/47.6.721 18192626 PMC2855242

[10] Sarkisian N , Gerstel N. Kin support among Blacks and whites: race and family organization. Am Sociol Rev. 2004;69(6):812–837. 10.1177/000312240406900604

[11] Silverstein M , Waite LJ. Are Blacks more likely than whites to receive and provide social support in middle and old age? Yes, no, and maybe so. J Gerontol. 1993;48(4):S212–S222. 10.1093/geronj/48.4.s212 8315245

[12] Janevic MR , Connell CM. Racial, ethnic, and cultural differences in the dementia caregiving experience: recent findings. Gerontologist. 2001;41(3):334–347. 10.1093/geront/41.3.334 11405431

[13] Parker LJ , Fabius C. Who’s helping whom? Examination of care arrangements for racially and ethnically diverse people living with dementia in the community. J Appl Gerontol. 2022;41(12):2589–2593. 10.1177/07334648221120247 35960528 PMC10348595

[14] Lines L , Wiener J. Racial and ethnic disparities in Alzheimer’s disease: a literature review. ASPE. 2014. https://aspe.hhs.gov/reports/racial-ethnic-disparities-alzheimers-disease-literature-review-0. Accessed January 28, 2024.

[15] Pinquart M , Sörensen S. Ethnic differences in stressors, resources, and psychological outcomes of family caregiving: a meta-analysis. Gerontologist. 2005;45(1):90–106. 10.1093/geront/45.1.90 15695420

[16] Scharlach AE , Kellam R, Ong N, Baskin A, Goldstein C, Fox PJ. Cultural attitudes and caregiver service use. J Gerontol Soc Work. 2006;47(1-2):133–156. 10.1300/J083v47n01_09 16901881

[17] Freedman VA , Agree EM, Seltzer JA, et al The changing demography of late-life family caregiving: a research agenda to understand future care networks for an aging U.S. population. Gerontologist. 2023;64:gnad036. 10.1093/geront/gnad036 PMC10825830 36999951

[18] Jiang S , Zuo W, Guo Z, Caswell H, Tuljapurkar S. How does the demographic transition affect kinship networks? Demograph Res. 2023;48:899–930. 10.4054/demres.2023.48.32

[19] Song X , Mare RD. Shared lifetimes, multigenerational exposure, and educational mobility. Demography. 2019;56(3):891–916. 10.1007/s13524-019-00772-8 31098951 PMC6823084

[20] Alburez-Gutierrez D , Mason C, Zagheni E. The “Sandwich Generation” Revisited: global demographic drivers of care time demands. Population Devel Rev. 2021;47(4):997–1023. 10.1111/padr.12436

[21] Song X , Campbell CD. Genealogical microdata and their significance for social science. Ann Rev Sociol. 2017;43(1):75–99. 10.1146/annurev-soc-073014-112157 34135542 PMC8204665

[22] Alburez-Gutierrez D , Barban N, Caswell H, Kolk M, Margolis R, Smith-Greenaway E, Song X, Verdery AM, Zagheni E. Kinship, demography, and inequality: review and key areas for future development. 2022. 10.31235/osf.io/fk7x9

[23] Daw J , Verdery AM, Margolis R. Kin count(s): educational and racial differences in extended kinship in the United States. Population Devel Rev. 2016;42(3):491–517. 10.1111/j.1728-4457.2016.00150.x PMC6711384 31456597

[24] Reyes AM , Schoeni RF, Freedman VA. National estimates of kinship size and composition among adults with activity limitations in the United States. Demograph Res. 2021;45:1097–1114. 10.4054/demres.2021.45.36 PMC9432774 36051489

[25] Verdery AM , Margolis R. Projections of White and Black older adults without living kin in the United States, 2015 to 2060. Proc Natl Acad Sci U S A. 2017;114(42):11109–11114. 10.1073/pnas.1710341114 28973934 PMC5651770

[26] Furstenberg FF. Kinship reconsidered: research on a neglected topic. J Marriage Fam. 2020;82(1):364–382. 10.1111/jomf.12628 34334811 PMC8321395

[27] Lin Z. Diversity and dynamics in care networks of older Americans. Socius. 2024;10:23780231231223906. 10.1177/23780231231223906

[28] Roberto KA , Savla J. Extended family caregivers for persons living with dementia. J Fam Nurs. 2022;28(4):396–407. 10.1177/10748407221115455 35960005 PMC10112257

[29] Wolff JL , Spillman BC, Freedman VA, Kasper JD. A national profile of family and unpaid caregivers who assist older adults with health care activities. JAMA Int Med. 2016;176(3):372–379. 10.1001/jamainternmed.2015.7664 PMC4802361 26882031

[30] Spillman BC , Favreault M, Allen EH. Family Structures and Support Strategies in the Older Population: Implications for Baby Boomers Issue Brief. Urban Institute. https://aspe.hhs.gov/reports/family-structures-support-strategies-older-population-implications-baby-boomers-issue-brief-0. Published 2020. Accessed January 28, 2024.

[31] Bengtson VL. Beyond the nuclear family: the increasing importance of multigenerational bonds. J Marriage Family. 2001;63(1):1–16. 10.1111/j.1741-3737.2001.00001.x

[32] Reed MN , Li L, Pesando LM, Harris LE, Furstenberg FF, Teitler JO. Communication with kin in the wake of the covid-19 pandemic. Socius. 2023;9:23780231231199388. 10.1177/23780231231199388 PMC10906743 38435742

[33] Kasper JD , Freedman VA, Spillman BC, Wolff JL. The disproportionate impact of dementia on family and unpaid caregiving to older adults. Health Affairs (Project Hope). 2015;34(10):1642–1649. 10.1377/hlthaff.2015.0536 26438739 PMC4635557

[34] Spillman BC , Freedman VA, Kasper JD, Wolff JL. Change over time in caregiving networks for older adults with and without dementia. J Gerontol B Psychol Sci Soc Sci. 2020;75(7):1563–1572. 10.1093/geronb/gbz065 31102533 PMC7424285

[35] 2023 Alzheimer’s Disease Facts and Figures. Alzheimer’s Dement. 2023;19(4):1598–1695. 10.1002/alz.13016 36918389

[36] Matthews KA , Xu W, Gaglioti AH, et al Racial and ethnic estimates of Alzheimer’s disease and related dementias in the United States (2015–2060) in adults aged ≥65 years. Alzheimer’s Dement. 2019;15(1):17–24. 10.1016/j.jalz.2018.06.3063 30243772 PMC6333531

[37] Zhu Y , Chen Y, Crimmins EM, Zissimopoulos JM. Sex, race, and age differences in prevalence of dementia in medicare claims and survey data. J Gerontol B Psychol Sci Soc Sci. 2021;76(3):596–606. 10.1093/geronb/gbaa083 32588052 PMC7887731

[38] Fabius CD , Wolff JL, Kasper JD. Race differences in characteristics and experiences of Black and White caregivers of older Americans. Gerontologist. 2020;60(7):1244–1253. 10.1093/geront/gnaa042 32400881 PMC7491434

[39] Liu R , Chi I, Wu S. Caregiving burden among caregivers of people with dementia through the lens of intersectionality. Gerontologist. 2022;62(5):650–661. 10.1093/geront/gnab146 34606599

[40] Seltzer JA. Family change and changing family demography. Demography. 2019;56(2):405–426. 10.1007/s13524-019-00766-6 30838537 PMC6450727

[41] Goodman LA , Keyfitz N, Pullum TW. Family formation and the frequency of various kinship relationships. Theor Popul Biol. 1974;5(1):1–27. 10.1016/0040-5809(74)90049-5 4818405

[42] Murphy M. Long-term effects of the demographic transition on family and kinship networks in Britain. Popul Dev Rev. 2011;37(Suppl 1):55–80. 10.1111/j.1728-4457.2011.00378.x 21280365

[43] Caswell H. The formal demography of kinship: a matrix formulation. Demograph Res. 2019;41:679–712. 10.4054/demres.2019.41.24

[44] Caswell H , Margolis R, Verdery A. The formal demography of kinship V: kin loss, bereavement, and causes of death. Demograph Res. 2023;49:1163–1200. 10.4054/demres.2023.49.41 PMC12369935 40851929

[45] Song X , Caswell H. The role of kinship in racial differences in exposure to unemployment. Demography. 2022;59(4):1325–1352. 10.1215/00703370-10057831 35730738

[46] Feng K , Song X, Caswell H. Population aging and dementia burden in China: a kinship perspective. Population Association of America 2023 Annual Meeting. 2023.

[47] Kolk M , Andersson L, Pettersson E, Drefahl S. The Swedish Kinship Universe: a demographic account of the number of children, parents, siblings, grandchildren, grandparents, aunts/uncles, nieces/nephews, and cousins using National Population Registers. Demography. 2023;60(5):1359–1385. 10.1215/00703370-10955240 37680176

[48] Williams I , Alburez-Gutierrez D, Song X, Caswell H. DemoKin: An R package to implement demographic matrix kinship models. https://cran.r-project.org/web/packages/DemoKin/readme/README.html. Accessed January 28, 2024.

[49] Song X , Campbell CD, Lee JZ. Ancestry matters: patrilineage growth and extinction. Am Sociol Rev. 2015;80(3):574–602. 10.1177/0003122415576516 27041745 PMC4813328

[50] Wolf DA. Kinship and family support in aging societies. https://pure.iiasa.ac.at/id/eprint/2780/1/WP-86-081.pdf. Published December 1986. Accessed January 28, 2024.

[51] Tu EJ-C , Freedman VA, Wolf DA. Kinship and family support in Taiwan: a microsimulation approach. Res Aging. 1993;15(4):465–486. 10.1177/0164027593154006

[52] Heuser RL. Fertility Tables for Birth Cohorts by Color. U.S. Department of Health, Education, and Welfare, Public Health Service, Health Resources Administration, National Center for Health Statistics; 1976.

[53] Hudomiet P , Hurd MD, Rohwedder S. Trends in inequalities in the prevalence of dementia in the United States. Proc Natl Acad Sci U S A. 2022;119(46):e2212205119. 10.1073/pnas.2212205119 36343247 PMC9674270

[54] Freedman VA , Kasper JD, Spillman BC, Plassman Brenda L. Short-term changes in the prevalence of probable dementia: an analysis of the 2011–2015 National Health and Aging Trends Study. J Gerontol B Psychol Sci Soc Sci. 2018;73(suppl_1):S48–S56. 10.1093/geronb/gbx144 29669099 PMC6018980

[55] Lennon JC , Aita SL, Bene VAD, et al Black and White individuals differ in dementia prevalence, risk factors, and symptomatic presentation. Alzheimer’s *Dement*. 2022;18(8):1461–1471. 10.1002/alz.12509 34854531 PMC9160212

[56] National Academies of Sciences, Engineering, and Medicine. Families Caring for an Aging America. Washington, DC: The National Academies Press; 2016. 10.17226/23606 27905704

[57] Administration for Community Living. 2022 National strategy to support family caregivers. http://acl.gov/CaregiverStrategy. Published 2022. Accessed January 28, 2024.

[58] Furstenberg FF , Harris LE, Pesando LM, Reed MN. Kinship practices among alternative family forms in western industrialized societies. J Marriage Fam. 2020;82(5):1403–1430. 10.1111/jomf.12712 34305172 PMC8294648

[59] Taylor RJ , Chatters LM, Woodward AT, Brown E. Racial and ethnic differences in extended family, friendship, fictive kin, and congregational informal support networks. Fam Relations. 2013;62(4):609–624. 10.1111/fare.12030 PMC4116141 25089067

[60] Taylor RJ , Chatters LM, Cross CJ. Taking diversity seriously: within-group heterogeneity in African American extended family support networks. J Marriage Fam. 2021;83(5):1349–1372. 10.1111/jomf.12783 34711997 PMC8547778

[61] Rote SM , Moon H. Racial/ethnic differences in caregiving frequency: does immigrant status matter? J Gerontol B Psychol Sci Soc Sci. 2018;73(6):1088–1098. 10.1093/geronb/gbw106 27573991

[62] Garcia MA , Diminich ED, Lu P, et al Caregiving for foreign-born older adults with dementia. J Gerontol B Psychol Sci Soc Sci. 2023;78(Suppl_1):S4–S14. 10.1093/geronb/gbac153 36409465 PMC10010468

