# Supplementary material for: Kinship and Care: Racial Disparities in Potential Dementia Caregiving in the United States From 2000 to 2060
Source: J Gerontol A Biol Sci Med Sci. 2024 Nov 7;79(Suppl 1):S32–41. doi: 10.1093/gerona/glae106 (PMC11542221; doi:10.1093/gerona/glae106)
Supplement: glae106_suppl_Supplementary_Material [file glae106_suppl_supplementary_material.docx]

Supplementary Materials

for

Kin and Care: Racial Disparities in Potential Dementia Caregiving in the U.S. from 2000 to 2060


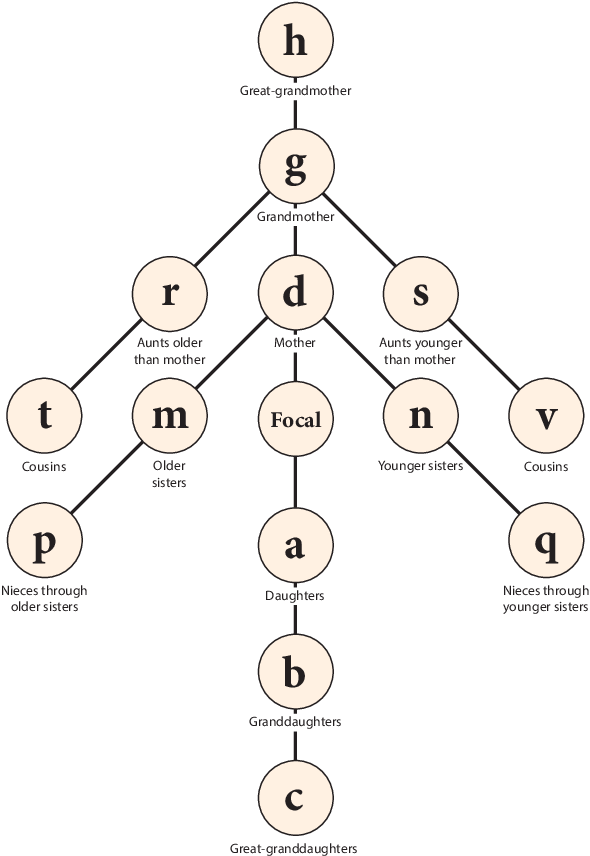


**eFigure 1. The kinship network from maternal lineage.**


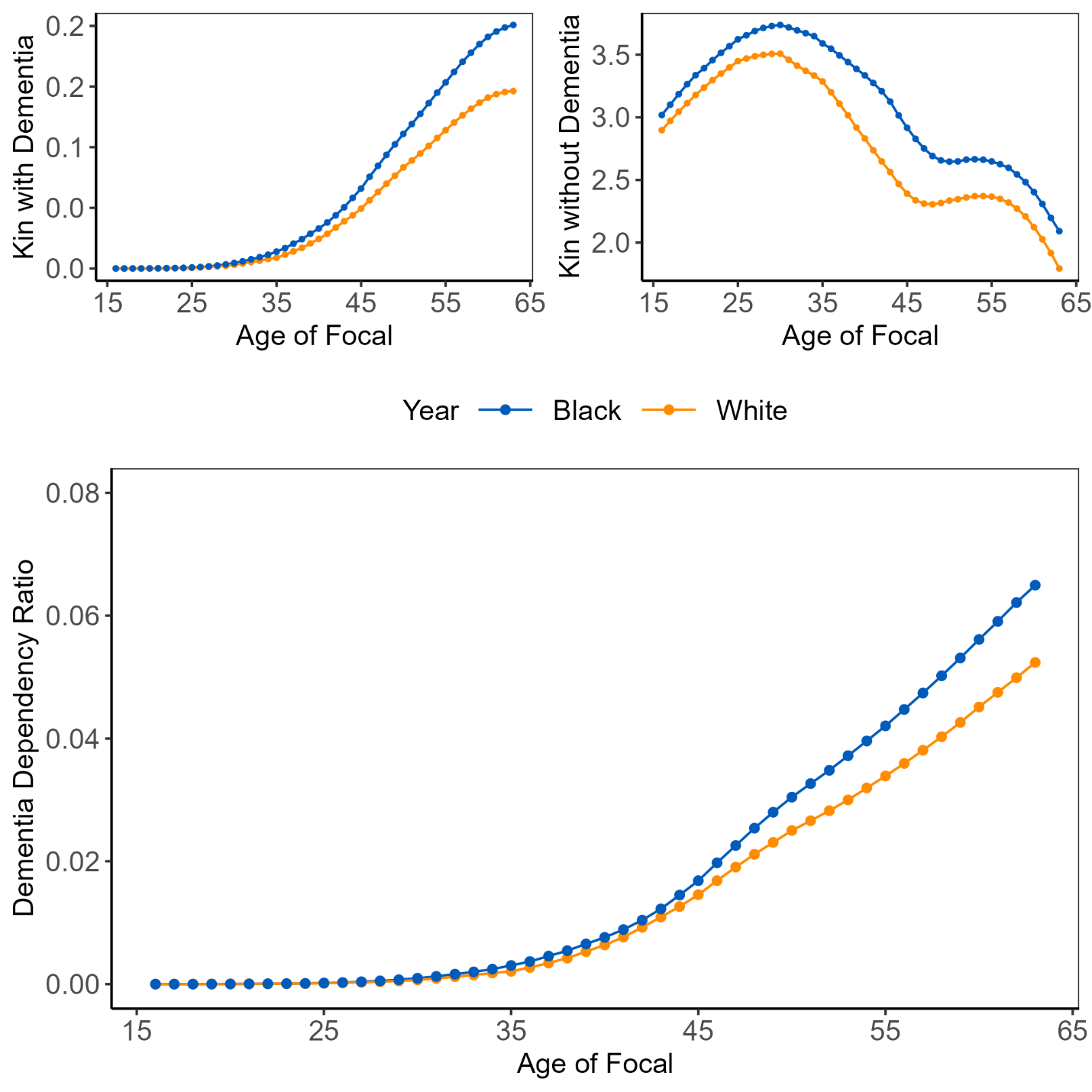


**eFigure 2. (A) The Number of Kin with Dementia by Race in 2016 (Including Only Parents and Children); (B) The Number of Kin Aged 16–64 Without Dementia by Race in 2016 (Including Only Parents and Children); (C) The Dementia Dependency Ratio as a Function of the Age of Focal by Race in 2016 (Including Only Parents and Children)**

*Note***:** We include only parents and children (siblings) in constructing the dementia dependency ratio.

*Sources*: National Vital Statistics Reports, 1996–2017; Vital Statistics of the United States, 1960–1995; Vital Statistics of the United States (abridged life table), 1946–1959; United States Life Tables and Actuarial Tables, 1939–1941; United States Life Tables for 1900–1902, 1901–1910, 1909–1911, 1919–1921, 1920–1929, and 1929–1931; Fertility Tables for Birth Cohorts by Color: United States, 1917–1980; National Vital Statistics Reports for 2015 and 2018; 2017 National Population Projections Datasets; Health and Retirement Study, 2000-2016 (Hudomiet et al. 2022).


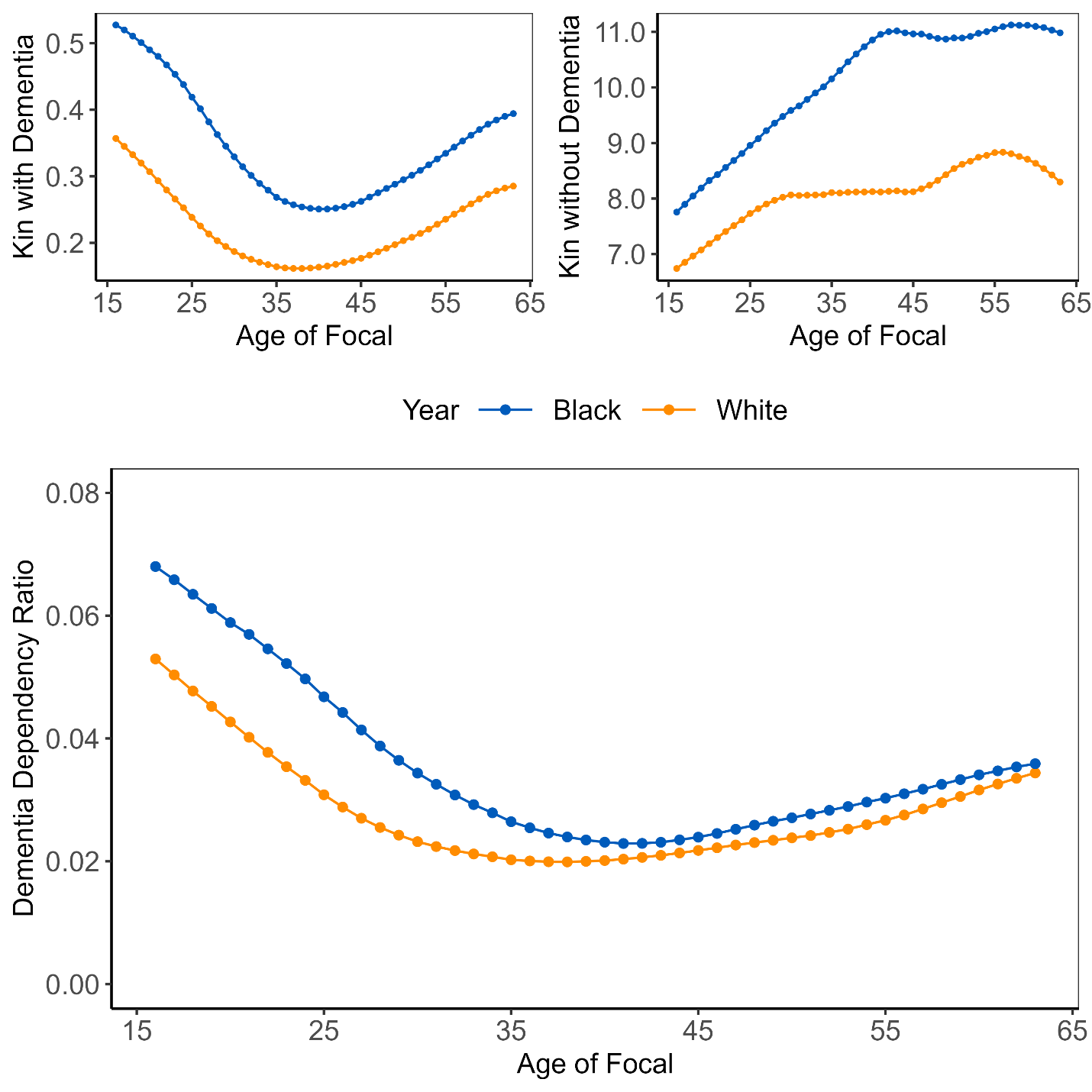


**eFigure 3. (A) The Number of Kin with Dementia by Race in 2016 (Reweighted); (B) The Number of Kin Aged 16–64 Without Dementia by Race in 2016 (Reweighted); (C) The Dementia Dependency Ratio as a Function of the Age of Focal by Race in 2016 (Reweighted)**

*Note***:** We keep all the kin types previously defined but assign a distinct weight to each based on genetic closeness to the Focal individual. For instance, parents, children, and siblings share half of their genes with the Focal, while aunts, uncles, nephews, nieces, grandparents, and grandchildren share a quarter of their genes with the Focal. Great-grandparents, great-grandchildren, and cousins share one-eighth of their genes with the Focal. Accordingly, we have allocated weights of 1, 0.5, and 0.25, respectively, to mirror the degree of closeness between the Focal and these various kin types.

*Sources*: National Vital Statistics Reports, 1996–2017; Vital Statistics of the United States, 1960–1995; Vital Statistics of the United States (abridged life table), 1946–1959; United States Life Tables and Actuarial Tables, 1939–1941; United States Life Tables for 1900–1902, 1901–1910, 1909–1911, 1919–1921, 1920–1929, and 1929–1931; Fertility Tables for Birth Cohorts by Color: United States, 1917–1980; National Vital Statistics Reports for 2015 and 2018; 2017 National Population Projections Datasets; Health and Retirement Study, 2000-2016 (Hudomiet et al. 2022).


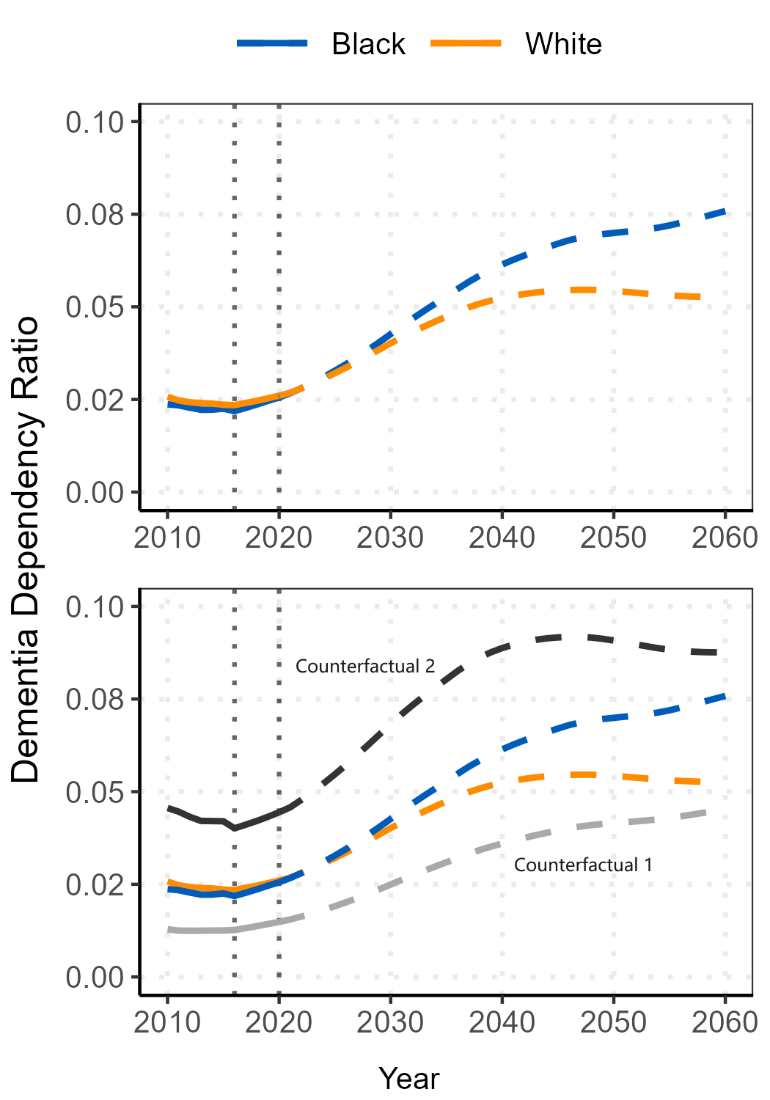


**eFigure 4. Estimated Dementia Dependency Ratio Between 2010 and 2020 and Projected Dementia Dependency Ratio From 2021 to 2060 (Including Only Parents and Children)**

*Sources*: National Vital Statistics Reports, 1996–2017; Vital Statistics of the United States, 1960–1995; Vital Statistics of the United States (abridged life table), 1946–1959; United States Life Tables and Actuarial Tables, 1939–1941; United States Life Tables for 1900–1902, 1901–1910, 1909–1911, 1919–1921, 1920–1929, and 1929–1931; Fertility Tables for Birth Cohorts by Color: United States, 1917–1980; National Vital Statistics Reports for 2015 and 2018; 2017 National Population Projections Datasets; Health and Retirement Study, 2000-2016 (Hudomiet et al. 2022).

*Note*: In Counterfactual 1, we maintained kinship differences but equalized dementia rates to match White individuals. In Counterfactual 2, we equalized kinship structures to the White, but let dementia rates vary by race. After the first vertical dashed line in 2016, dementia rates reflect the observed values observed in 2016. Beyond the second dashed line in 2020, the vital statistics are based on projections.

We include only parents and children (siblings) in constructing the dementia dependency ratio.


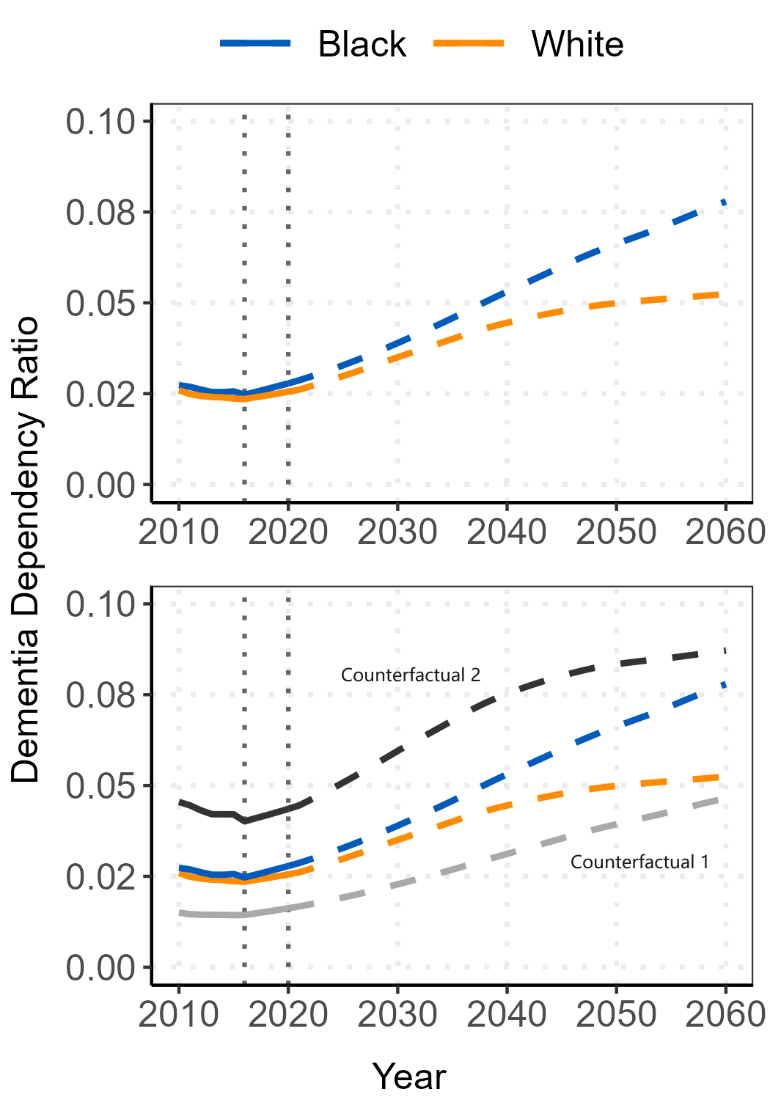


**eFigure 5. Estimated Dementia Dependency Ratio Between 2010 and 2020 and Projected Dementia Dependency Ratio From 2021 to 2060 (Reweighted)**

*Sources*: National Vital Statistics Reports, 1996–2017; Vital Statistics of the United States, 1960–1995; Vital Statistics of the United States (abridged life table), 1946–1959; United States Life Tables and Actuarial Tables, 1939–1941; United States Life Tables for 1900–1902, 1901–1910, 1909–1911, 1919–1921, 1920–1929, and 1929–1931; Fertility Tables for Birth Cohorts by Color: United States, 1917–1980; National Vital Statistics Reports for 2015 and 2018; 2017 National Population Projections Datasets; Health and Retirement Study, 2000-2016 (Hudomiet et al. 2022).

*Note*: In Counterfactual 1, we maintained kinship differences but equalized dementia rates to match White individuals. In Counterfactual 2, we equalized kinship structures to the White, but let dementia rates vary by race. After the first vertical dashed line in 2016, dementia rates reflect the observed values observed in 2016. Beyond the second dashed line in 2020, the vital statistics are based on projections.

We keep all the kin types previously defined but assign a distinct weight to each based on genetic closeness to the Focal individual. For instance, parents, children, and siblings share half of their genes with the Focal, while aunts, uncles, nephews, nieces, grandparents, and grandchildren share a quarter of their genes with the Focal. Great-grandparents, great-grandchildren, and cousins share one-eighth of their genes with the Focal. Accordingly, we have allocated weights of 1, 0.5, and 0.25, respectively, to mirror the degree of closeness between the Focal and these various kin types.


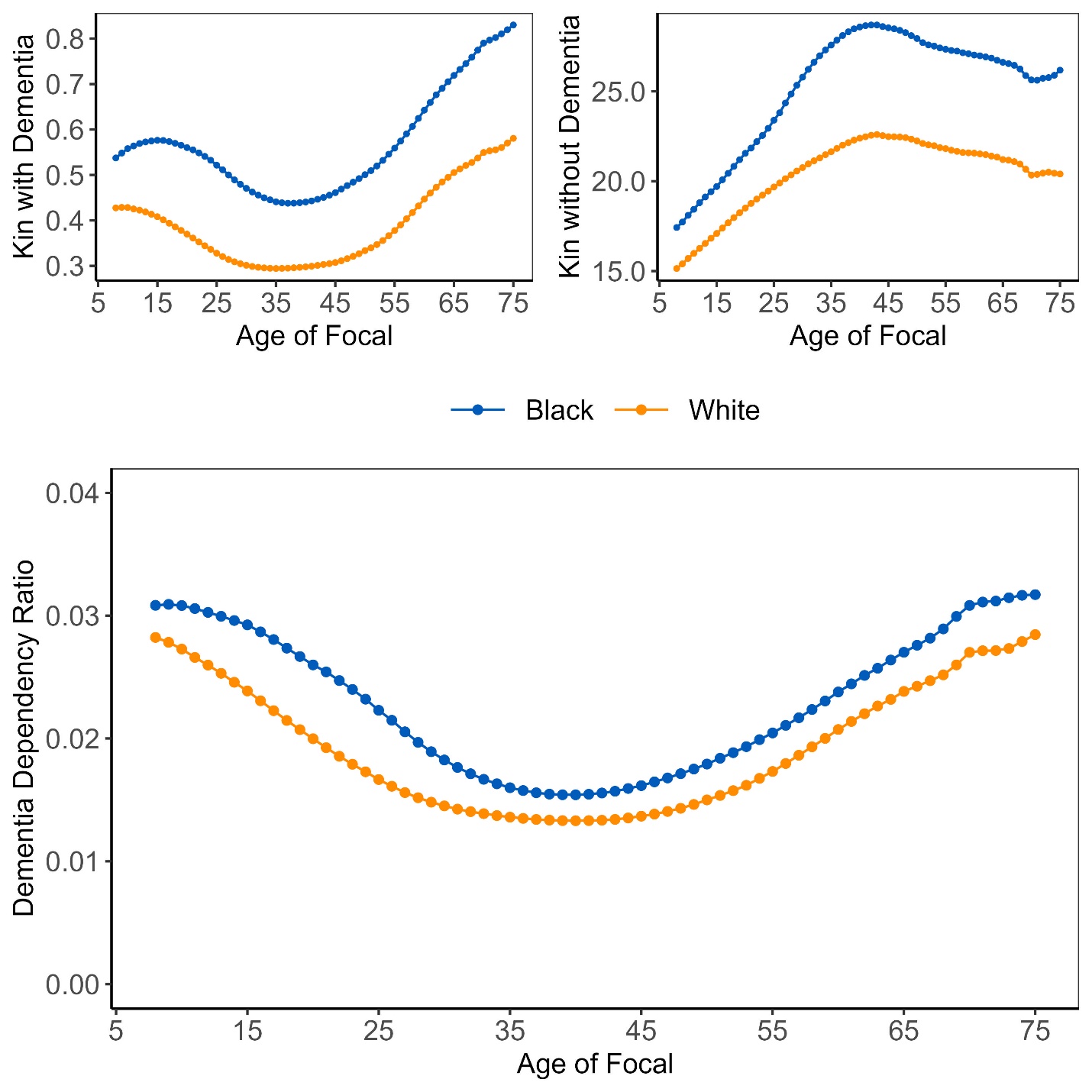
 **eFigure 6. (A) The Number of Kin with Dementia by Race in 2016; (B) The Number of Kin Aged 8–75 Without Dementia by Race in 2016; (C) The Dementia Dependency Ratio as a Function of the Age of Focal by Race in 2016**

*Note***:** We include only parents and children (siblings) in constructing the dementia dependency ratio.

*Sources*: National Vital Statistics Reports, 1996–2017; Vital Statistics of the United States, 1960–1995; Vital Statistics of the United States (abridged life table), 1946–1959; United States Life Tables and Actuarial Tables, 1939–1941; United States Life Tables for 1900–1902, 1901–1910, 1909–1911, 1919–1921, 1920–1929, and 1929–1931; Fertility Tables for Birth Cohorts by Color: United States, 1917–1980; National Vital Statistics Reports for 2015 and 2018; 2017 National Population Projections Datasets; Health and Retirement Study, 2000-2016 (Hudomiet et al. 2022).

In our main results, we use the age range from 16 to 64 based on a recent report indicating that the majority of caregivers fall within this age range. It may overlook caregiving among youth and older caregivers. Here, we extends the age range in the denominator to 8 and 75. This age range is supported by the following findings: (1) According to a 2020 report from the National Alliance for Caregiving and AARP, the average age of caregivers in the US is 49.4 years old. 82\% of caregivers are between 18 and 64 years old, while 12\% are between 65 and 74 years old. (2) A 2005 report by the National Alliance for Caregiving states that there are at least 1.3 million caregiving youth between the ages of 8 and 18 in the United States.


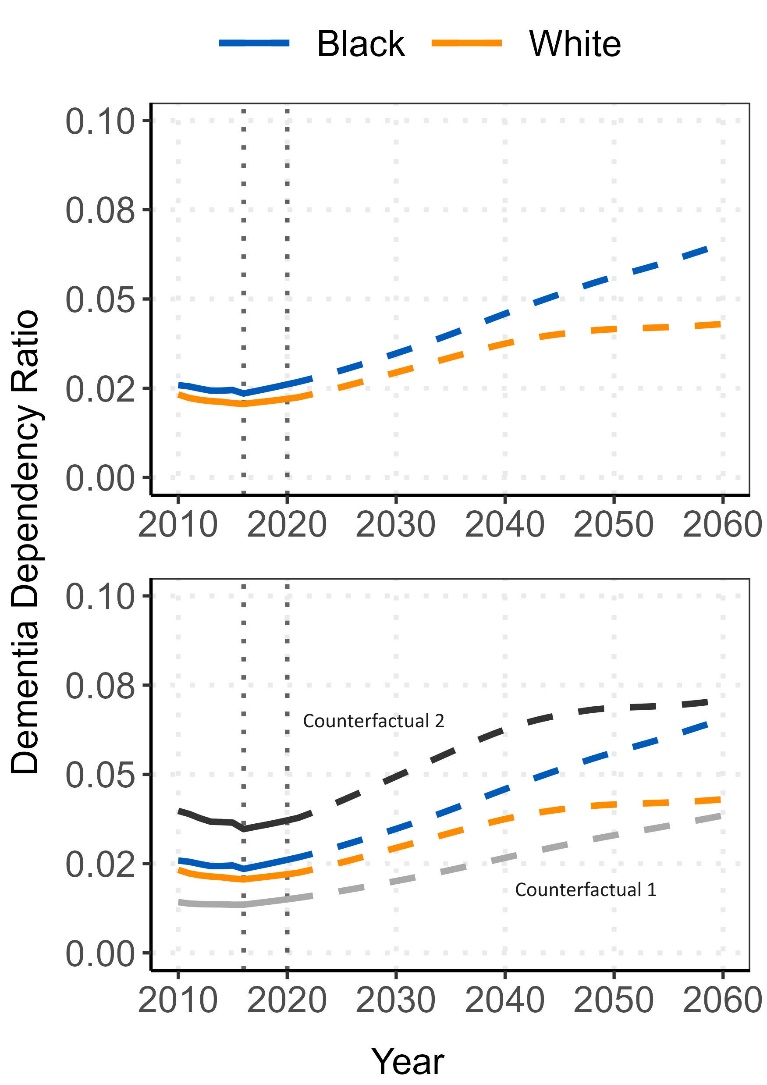


**eFigure 7. Estimated Dementia Dependency Ratio Between 2010 and 2020 and Projected Dementia Dependency Ratio From 2021 to 2060 (Aged 8-75)**

*Sources*: National Vital Statistics Reports, 1996–2017; Vital Statistics of the United States, 1960–1995; Vital Statistics of the United States (abridged life table), 1946–1959; United States Life Tables and Actuarial Tables, 1939–1941; United States Life Tables for 1900–1902, 1901–1910, 1909–1911, 1919–1921, 1920–1929, and 1929–1931; Fertility Tables for Birth Cohorts by Color: United States, 1917–1980; National Vital Statistics Reports for 2015 and 2018; 2017 National Population Projections Datasets; Health and Retirement Study, 2000-2016 (Hudomiet et al. 2022).

*Note*: In Counterfactual 1, we maintained kinship differences but equalized dementia rates to match White individuals. In Counterfactual 2, we equalized kinship structures to the White, but let dementia rates vary by race. After the first vertical dashed line in 2016, dementia rates reflect the observed values observed in 2016. Beyond the second dashed line in 2020, the vital statistics are based on projections.
